# Abnormal Default-Mode Network Homogeneity in Melancholic and Nonmelancholic Major Depressive Disorder at Rest

**DOI:** 10.1155/2021/6653309

**Published:** 2021-04-26

**Authors:** Meiqi Yan, Xilong Cui, Feng Liu, Huabing Li, Renzhi Huang, Yanqing Tang, Jindong Chen, Jingping Zhao, Guangrong Xie, Wenbin Guo

**Affiliations:** ^1^National Clinical Research Center for Mental Disorders, and Department of Psychiatry, The Second Xiangya Hospital of Central South University, Changsha, 410011 Hunan, China; ^2^Department of Radiology, Tianjin Medical University General Hospital, Tianjin 300000, China; ^3^Department of Radiology, The Second Xiangya Hospital of Central South University, Changsha, 410011 Hunan, China; ^4^Hunan Key Laboratory of Children's Psychological Development and Brain Cognitive Science, Changsha, 410205 Hunan, China; ^5^Department of Psychiatry, The First Affiliated Hospital of China Medical University, Shenyang, Liaoning 110001, China; ^6^Department of Psychiatry, The Third People's Hospital of Foshan, Foshan, Guangdong 528000, China

## Abstract

**Background:**

Melancholic depression has been assumed as a severe type of major depressive disorder (MDD). We aimed to explore if there were some distinctive alterations in melancholic MDD and whether the alterations could be used to discriminate the melancholic MDD and nonmelancholic MDD.

**Methods:**

Thirty-one outpatients with melancholic MDD, thirty-three outpatients with nonmelancholic MDD, and thirty-two age- and gender-matched healthy controls were recruited. All participants were scanned by resting-state functional magnetic resonance imaging (fMRI). Imaging data were analyzed with the network homogeneity (NH) and support vector machine (SVM) methods.

**Results:**

Both patient groups exhibited increased NH in the right PCC/precuneus and right angular gyrus and decreased NH in the right middle temporal gyrus compared with healthy controls. Compared with nonmelancholic patients and healthy controls, melancholic patients exhibited significantly increased NH in the bilateral superior medial frontal gyrus and decreased NH in the left inferior temporal gyrus. But merely for melancholic patients, the NH of the right middle temporal gyrus was negatively correlated with TEPS total and contextual anticipatory scores. SVM analysis showed that a combination of NH values in the left superior medial frontal gyrus and left inferior temporal gyrus could distinguish melancholic patients from nonmelancholic patients with accuracy, sensitivity, and specificity of 79.66% (47/59), 70.97% (22/31), and 89.29%(25/28), respectively.

**Conclusion:**

Our findings showed distinctive network homogeneity alterations in melancholic MDD which may be potential imaging markers to distinguish melancholic MDD and nonmelancholic MDD.

## 1. Introduction

Major depression disorder (MDD), a common psychiatric disabling disease, ranks the ninth killer of diseases [1]. More than 350 million people in the world suffer from it for many years [1]. Melancholic depression has been assumed as a severe type of MDD for its clinical features. Despite the common characteristics of MDD, like decreased reactivity of mood and affect, a pervasive and pronounced depressive mood that is especially worse in the morning, melancholic depression is also characterized by pervasive anhedonia, guilt, psychomotor disturbance (like retardation and spontaneous agitation), cognitive impairment (like reduced concentration and impaired working memory), and vegetative dysfunction (like interrupted sleep, appetite, and weight loss), and these features have great consistency [2, 3]. Although it is still unclear whether melancholic depression is a distinct mental disorder or merely a putative subtype of MDD [4], an obviously different and more impaired performance on cognition was observed in melancholic depression, particularly on processing speed, problem solving, and visual learning [5]. Besides, the melancholic patients require longer periods for cognitive recovery relative to the nonmelancholic individuals [6]. Melancholic depression generally responds to pharmacotherapy and electroconvulsive therapy better [3]. Major depression with melancholic features shows different outcome patterns for antidepressants [7]. Thus, there will be superior predictive validity for patients' treatments and prognoses because of definite diagnosis of melancholic depression [2].

Many previous studies were aimed at exploring the neurobiological and neurochemical mechanisms of the melancholic MDD in order to distinguish the two subtypes of MDD [8–10]. Patients with melancholic MDD exhibited lower NH values in the right middle temporal gyrus as well as the temporal pole (MTG/TP) than healthy controls [11]. Patients with melancholic MDD also showed reduced mean fractional anisotropy (FA) in the right ventral tegmental area-lateral orbitofrontal cortex (VTA-lOFC) connection compared with patients with nonmelancholic MDD [12]. And our previous study reported disrupted regional homogeneity (ReHo) in melancholic and nonmelancholic MDD [13]. However, it still remains unknown whether melancholic MDD is specific or significantly different from nonmelancholic MDD in the terms of network homogeneity.

Default-mode network (DMN), composed of a distributed network of brain regions including the medial prefrontal cortex (MPFC), posterior cingulate cortex/precuneus (PCC/PCu), bilateral temporal cortices, and inferior parietal cortex, is getting increasing attention in the study of mental disorders. Many previous studies have already revealed the important role that DMN may play in etiological and pathological mechanisms of psychiatric disorders [11, 14–17]. But the findings of the abnormal DMN involved in the neurophysiology of MDD were inconsistent. Greater functional connectivity (FC) was found in the subgenual cingulate and thalamus in MDD, and FC in the area of the subgenual cingulate was positively correlated with the duration of the current depressive episode [18]. Increased connectivity to the bilateral dorsal medial prefrontal cortex region was observed in MDD [19]. Both increased and decreased NH in the DMN regions were also observed in first-episode drug-naive MDD [17]. Patients with melancholic MDD exhibited lower NH values in the right middle temporal gyrus as well as the temporal pole (MTG/TP) [11]. The inconsistent findings can be attributed to different samples and methods. Their enrolled patients owned different illness durations and inclusion/exclusive criteria, and their scanner parameters were also discrepant. Especially for the melancholic MDD study [11], nonmelancholic patients were not enrolled. Because MDD has many different subtypes, to make the results more comparative and practical, we could recruit melancholic patients, nonmelancholic patients, and healthy controls, and then we might find some distinctive and unique features of melancholic MDD.

In the present study, the network homogeneity (NH) approach was used to explore DMN homogeneity in patients with melancholic MDD and nonmelancholic MDD. We aimed to explore if there were some distinctive alterations in melancholic MDD and whether the alterations could be used to discriminate the melancholic MDD and nonmelancholic MDD. We hypothesized that melancholic MDD patients had NH alterations in the DMN regions and the altered regions might be distinct areas that could be applied to separate melancholic MDD from nonmelancholic MDD. We also hypothesized that abnormal NH would be related to the clinical features in the patients.

## 2. Methods

### 2.1. Participants

We recruited 31 patients with melancholic MDD and 33 patients with nonmelancholic MDD, aged from 18 to 45 years old. They were all from the outpatients of the Second Xiangya Hospital of Central South University, China. The data were collected from May 4, 2014, to December 30, 2016. The diagnosis was determined independently by two psychiatrists, according to the Diagnostic and Statistical Manual of Mental Disorders, Fourth Edition (DSM-IV) [20]. All the patients must meet the following inclusion criteria: (1) first major depressive episode, accessed by the Hamilton Rating Scale for Depression (HRSD-17) [21] with a total HRSD scores of ≥17; (2) an illness duration < 12 months; (3) drug-naive and with no history of electroconvulsive therapy. The required criteria for MDD with melancholic features in DSM-IV were as follows: (1) at least one of the two following symptoms presents at the most severe stage of the current episode: pervasive anhedonia and/or nonreactive mood; (2) three (or more) of the following symptoms: characteristic depressive mood, regularly worse in the morning, early morning awakening, marked psychomotor agitation or retardation, significant anorexia or weight loss, and excessive or inappropriate guilt. The nonmelancholic MDD group was made up of patients who did not meet these criteria, that is, (1) meet the uniform patient inclusion criteria described above and (2) with none of the above melancholic traits.

We also recruited 32 age- and gender-matched healthy controls from the community. None of them were related to the patients, and none had a family history of mental illness, especially their first degree relatives. Besides, they would also be excluded if they had any history of neurological disorders, substance abuse, or psychiatric symptoms.

All the participants would be excluded if they met the following criteria: (1) other mental disorders that meet the DSM-IV diagnostic criteria; (2) a history of neurological disorders, any serious physical illnesses, and substance abuse; (3) initial MRI scan showed brain structural abnormalities; (4) pregnancy; (5) any contraindications to MRI scanning.

All participants were Han Chinese and right-handed, with no less than 9 years of education. The depressive severity was assessed by using the HRSD-17, the Chinese version of the Snaith-Hamilton Pleasure Scale (SHAPS-C) [22] was administered to evaluate anhedonic states, and the Beck anxiety inventory (BAI) [23] was applied to evaluate anxiety state for all subjects. The Chinese version of the Temporal Experience of Pleasure Scale (TEPS) [24] was used to capture the anticipatory and consummatory facets of pleasure.

The study was approved by the Medical Research Ethics Committee of the Second Xiangya Hospital of Central South University, China. The study was conducted in accordance with the Helsinki Declaration. Each participant has completed an informed consent prior to enrollment.

### 2.2. Image Acquisition

Resting-state fMRI data were obtained using a 3.0 T Siemens scanner (Germany) at the Second Xiangya Hospital of Central South University. All the subjects were instructed to lay supine, close their eyes, and stay still and awake. The resting-state functional images were acquired with echo planar imaging (EPI) sequence by the following parameters: repetition time/echo time (TR/TE) 2500/25 ms, 39 slices, 64∗64 matrix, 90° flip angle, 24 cm field of view, 3.5 mm slice thickness, no gap, and 200 volumes lasting for 500 s.

### 2.3. Data Preprocessing

The Data Processing Assistant for Resting-State fMRI (DPARSF) was used for data preprocessing in MATLAB (MathWorks) [25]. To reduce the potential influences from initial instability of MRI signal and adaptation period of patients, the first 10 images were discarded. After the correction for slice timing and head motion, the participants with maximum displacement of *x*, *y*, or *z* axis exceeding 2 mm or more than 2° of maximum angular rotation would be excluded. Then, the corrected imaging data were spatially normalized to the MNI space and got resampled to 3 × 3 × 3 mm^3^. After that, the generated imaging data were temporally bandpass filtered (0.01–0.08 Hz) and got linearly detrended. Several spurious covariates were also removed from the images, such as the signal from the ventricular seed-based region of interest (ROI) and the white matter-centered region, as well as the 24 head motion parameters acquired by rigid body correction. The global signal was still preserved while preprocessing the resting-state FC data as suggested by the previous study [26].

### 2.4. DMN Identification

Each participant was analyzed using group independent component analysis (ICA) method. The analysis included three main steps as follows: data reduction, independent component (IC) separation, and back reconstruction. The DMN components for each group were selected and picked out based on the templates provided by GIFT. Then, the DMN components were overlaid to generate the DMN mask in the following NH analysis, similar to our previous research [11].

### 2.5. NH Analysis

NH analysis was conducted by MATLAB (MathWorks). For each subject, correlation coefficients were calculated between each voxel and all other voxels within the DMN mask. The mean correlation coefficient was defined as the homogeneity of the given voxel. The average NH of each voxel in the DMN mask was generated. The averaged NH maps were smoothed with a Gaussian kernel of 4 mm full-width at half-maximum [27]. The NH maps within the DMN mask were applied for the comparisons between groups.

### 2.6. Statistical Analyses

The analysis of variance (ANOVA) approach was performed in SPSS19.0 software (LSD between two group comparisons) to analyze whether there were differences across the three groups in age, years of education and HRSD-17, BAI, and SHAPS-C scores. We applied a two-sample *t*-test to analyze and compare group differences in the illness duration and TEPS scores between melancholic MDD and nonmelancholic MDD patient groups. Gender distributions were compared by using a chi-square test. The significance level was *p* < 0.05.

NH analysis was performed with analysis of covariance (ANCOVA) across the three groups. Then, post hoc *t*-tests were executed to compare NH differences between every two groups. Using Gaussian Random Field (GRF) theory, the significance threshold for multiple comparisons was set at *p* < 0.05 (voxel significance: *p* < 0.001, cluster significance: *p* < 0.05). The FC at rest can be affected by the micromotion from one volume to another, so we calculated the framewise displacement (FD) value for each subject according to the previous study [28]. Age, years of education, and average FD values were used as covariates for NH comparisons between groups to minimize the potential impact of these variables.

### 2.7. Correlation Analyses

The average NH values were extracted from the brain clusters with abnormal NH values. Pearson's correlation analysis was applied to determine the correlations between abnormal NH and HRSD-17, BAI, SHAPS-C, and TEPS scores. Benjamini-Hochberg correction threshold value *p* < 0.05 was adopted.

### 2.8. Classification Analyses

The support vector machine (SVM) approach was applied to test the feasibility and effectiveness of using the NH values identified in the abnormal brain regions to distinguish melancholic MDD and nonmelancholic MDD by conducting the LIBSVM software package (http://www.csie.ntu.edu.tw/~cjlin/libsvm/) in MATLAB. In the study, the method of “leave-one-out” was applied.

## 3. Results

### 3.1. Demographic Characteristics and Clinical Information

On account of excessive head movement, 5 patients with nonmelancholic MDD were excluded. Finally, 31 patients with melancholic MDD, 28 patients with nonmelancholic MDD, and 32 healthy controls were included in the analyses. No significant age and gender differences were observed across the three groups, and the illness duration did not significantly differ between the two patient groups. There were significant differences among the three groups in years of education, HRSD-17 scores, BAI scores, and SHAPS-C scores. The educational level of the nonmelancholic MDD group was significantly lower than that of the melancholic MDD group (*p* = 0.001) and the healthy control group (*p* = 0.01). HRSD-17 scores, BAI scores, and SHAPS-C scores of the melancholic MDD group (*p* < 0.001) and nonmelancholic MDD group (*p* < 0.001) were significantly higher than those of the healthy control group. Significantly higher BAI (*p* = 0.024) and SHAPS-C scores (*p* < 0.001) of the melancholic group were observed than those of the nonmelancholic group, while HRSD-17 scores showed no significant difference between the two groups (*p* = 0.743). Significant differences of the partial TEPS scores between the two patient groups were also observed. The melancholic group showed significantly lower TEPS total scores (*p* = 0.002), TEPS abstract anticipatory scores (*p* = 0.001), and TEPS contextual anticipatory scores (*p* = 0.001) than the nonmelancholic group. No significant difference was observed in the TEPS abstract consummatory scores (*p* = 0.117) and TEPS contextual consummatory scores (*p* = 0.074) between the two patient groups. More details of demographic and clinical data are presented in Table 1.

### 3.2. NH Differences between Groups

**Figure 1:**
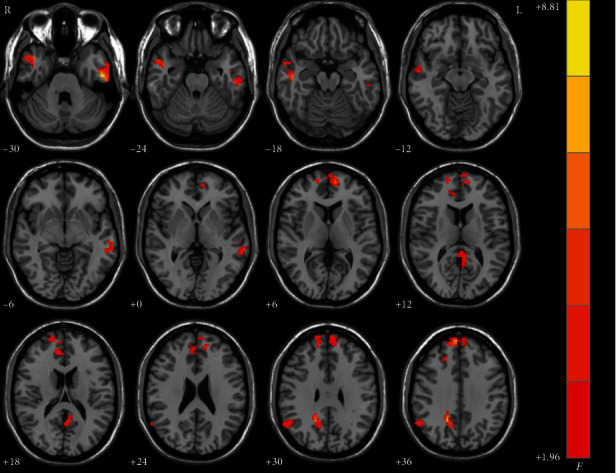
Brain regions within the DMN showing group differences of NH values among three groups. Color bar indicates *F* values from ANCOVA (age, years of education, and framewise displacement as covariates). NH: network homogeneity; DMN: default mode network; ANCOVA: analysis of covariance.

Figure 1 showed the brain regions within the DMN showing group differences of NH across the three groups by using ANCOVA. The group differences were mainly in the frontal, temporal, parietal, and limbic regions of the DMN.

#### 3.2.1. Melancholic vs. Healthy Controls

**Figure 2:**
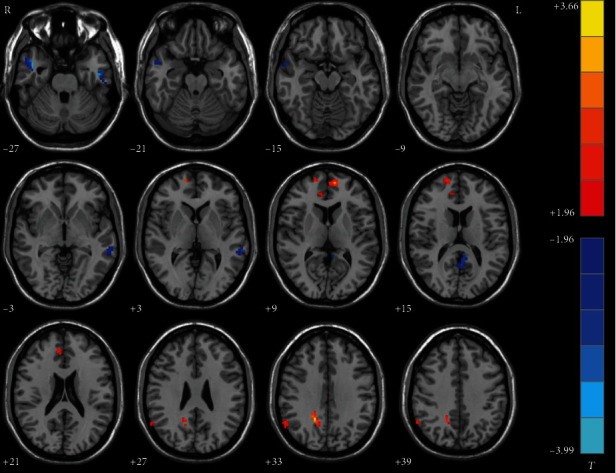
Statistical map depicts higher and lower NH of melancholic patients compared with healthy controls. The threshold was set at *p* < 0.05. Blue denotes lower NH and red denotes higher NH. Color bar indicates *T* values from two-sample *t*-test. L: left side; R: right side; DMN: default mode network; NH: network homogeneity.

Compared with healthy controls, melancholic MDD patients showed significantly lower NH in the left inferior temporal gyrus, bilateral middle temporal gyrus, and left PCC/precuneus but higher NH in the bilateral superior medial frontal gyrus, right anterior cingulate cortex, right angular gyrus, and right PCC/precuneus (Table 2, Figure 2).

#### 3.2.2. Nonmelancholic vs. Healthy Controls

**Figure 3:**
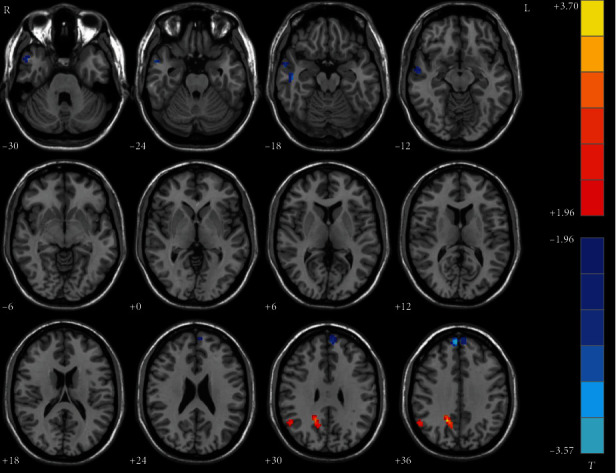
Statistical map depicts higher and lower NH of nonmelancholic patients compared with healthy controls. The threshold was set at *p* < 0.05. Blue denotes lower NH and red denotes higher NH. Color bar indicates *T* values from two-sample *t*-test. L: left side; R: right side; DMN: default mode network; NH: network homogeneity.

Compared with healthy controls, nonmelancholic MDD patients showed significantly lower NH in the right middle temporal gyrus and bilateral superior medial frontal gyrus but higher NH in the right PCC/precuneus and right angular gyrus (Table 2, Figure 3).

#### 3.2.3. Melancholic vs. Nonmelancholic

**Figure 4:**
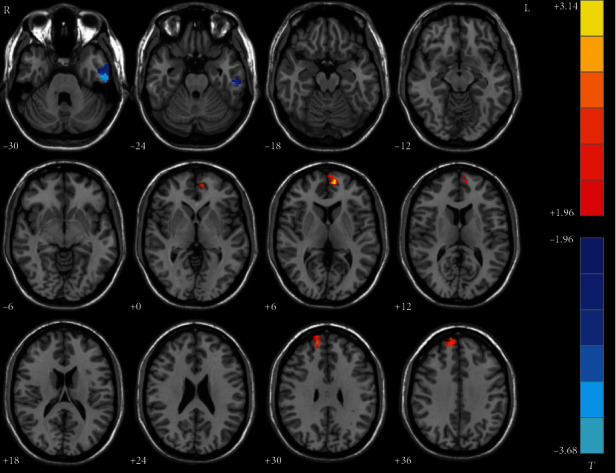
Statistical map depicts higher and lower NH of melancholic patients compared with nonmelancholic patients. The threshold was set at *p* < 0.05. Blue denotes lower NH and red denotes higher NH. Color bar indicates *T* values from two-sample *t*-test. L: left side; R: right side; DMN: default mode network; NH: network homogeneity.

The melancholic MDD patients showed decreased NH in the left inferior temporal gyrus and increased NH in the bilateral superior medial frontal gyrus compared with the nonmelancholic MDD patients (Table 2, Figure 4).

### 3.3. Correlations between NH and Clinical Characteristics

**Figure 5:**
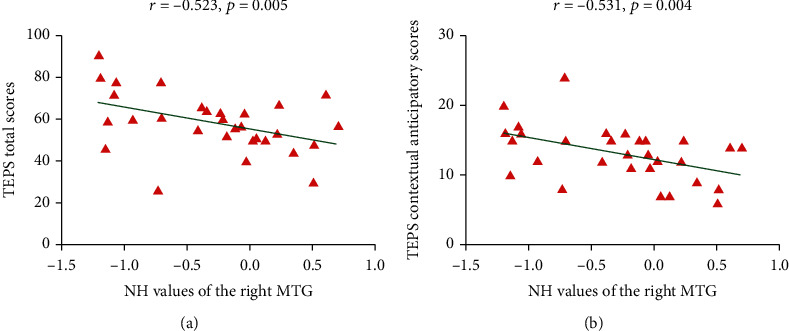
(a) Correlation between the NH values of the right middle temporal gyrus and the TEPS total scores in melancholic patients. (b) Correlation between the NH values of the right middle temporal gyrus and the TEPS contextual anticipatory scores in melancholic patients.

There were no significant correlations between NH and clinical characteristics in the nonmelancholic patients. For the melancholic patients, the NH values of the right middle temporal gyrus, which differed in different brain regions between patients and healthy controls, was negatively correlated with TEPS total scores (*r* = −0.523, *p* = 0.005, Benjamini-Hochberg correction *p* = 0.045) and TEPS contextual anticipatory scores (*r* = −0.531, *p* = 0.004, Benjamini-Hochberg correction *p* = 0.036) (Figure 5).

### 3.4. Discriminating Patients with Melancholic MDD from Nonmelancholic MDD

**Figure 6:**
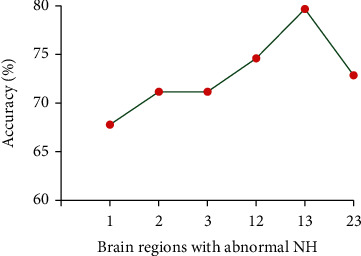
The accuracy of using abnormal NH values in different brain regions to classify patients. 1: left superior medial frontal gyrus; 2: right superior medial frontal gyrus; 3: left inferior temporal gyrus; 12: left superior medial frontal gyrus and right superior medial frontal gyrus; 13: left superior medial frontal gyrus and left inferior temporal gyrus; 23: right superior medial frontal gyrus and left inferior temporal gyrus.

**Figure 7:**
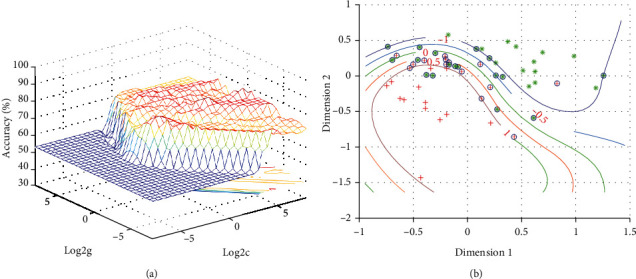
Visualization of classifications through support vector machine (SVM) using the combination of the NH values in the left superior medial frontal gyrus and left inferior temporal gyrus. (a) SVM parameter result of 3D view. (b) Dimension 1 and dimension 2 represent the NH values in the left superior medial frontal gyrus and left inferior temporal gyrus, respectively. Red crosses represent nonmelancholic MDD patients, and green crosses represent the melancholic MDD patients. MDD: major depressive disorder.

Figure 6 presents the general information of SVM results for discriminating patients with melancholic MDD from nonmelancholic MDD. The result showed that the abnormal NH values in the combination region of the left superior medial frontal gyrus and left inferior temporal gyrus exhibited the highest accuracy (Figure 6), with an accuracy of 79.66% (47/59), a sensitivity of 70.97% (22/31), and a specificity of 89.29% (25/28) for distinguishing patients with melancholic MDD and patients with nonmelancholic MDD (Figure 7).

## 4. Discussion

In the present study, we investigated the DMN homogeneity in patients with melancholic MDD and patients with nonmelancholic MDD at rest. We found that both patients with melancholic and nonmelancholic MDD exhibited increased NH values in the right PCC/precuneus and right angular gyrus (AG) and decreased NH values in the right middle temporal gyrus (MTG) compared with healthy controls. Compared with nonmelancholic MDD patients and healthy controls, melancholic patients exhibited significant increased NH values in the bilateral superior medial frontal gyrus (SMFG) and decreased NH values in the left inferior temporal gyrus (ITG). But merely for melancholic patients, the NH value of the right MTG was negatively correlated with TEPS total scores and TEPS contextual anticipatory scores. In addition, the SVM analysis showed that a combination of the NH values in the left SMFG and left ITG might be employed as a potential imaging marker to distinguish melancholic MDD patients from nonmelancholic MDD patients.

PCC/precuneus, one adjacent part of the posterior DMN, plays an important role in memory and problem-solving task processes [29, 30]. In some previous studies, decreased activity has been observed in the PCC/precuneus. PCC/precuneus showed decreased FC [31–33] as well as decreased voxel-mirrored homotopic connectivity (VMHC) [34, 35]. And decreased regional homogeneity (ReHo) [36] was also observed in the PCC/precuneus. But some other previous studies have revealed increased activity in the PCC/precuneus. Precuneus showed higher FC in MDD compared with healthy controls at rest [19, 37]. Consistent with the previous findings of hyperactivity in PCC/precuneus, we observed increased NH values in the right PCC/precuneus in the melancholic and nonmelancholic MDD patients in the present study, compared with healthy controls. Several confounding factors, such as illness durations, medication use, different methods, and scanner parameters, might be relevant to the inconsistent findings, so we could not compare them directly. But certainly, our results highlighted the important role of the PCC/precuneus in the pathophysiology in MDD. Impaired memory was seen as one of the most common cognitive disturbances in MDD, especially the episodic autobiographical memory [38, 39]. And the AG might be the part of the brain networks that participated in episodic autobiographical memory and is attributed to producing the subjective experience of remembering [40, 41] and had as a critical role in conceptual combination [42]. We observed that both melancholic and nonmelancholic MDD patients exhibited higher NH values in the right AG than healthy controls, which might interpret the memory impairment in MDD. Decreased correlation strength of gray matter volume [43] and FC [44] was found between the right AG and PCC in MDD. Although we did not explore whether MDD exhibited structural or functional correlation alterations between the PCC and the right AG in the present study, we could speculate that there might be some structural and functional correlation alterations between the PCC and the right AG in MDD.

In previous studies, the temporal lobe has been illustrated to be involved in emotional regulation, process of memory, and social cognition [45, 46]. In the present study, both melancholic and nonmelancholic patients exhibited lower NH values in the right MTG than healthy controls, which was consistent with previous studies that the temporal gyrus showed lower NH values in the patients with melancholic MDD compared with healthy controls [11] and that the MDD patients showed abnormal FC and decreased regional activity in temporal regions [47–50]. That might be able to interpret the emotional regulation impairment and memory deficit in MDD. As mentioned above, we speculated that abnormal NH in the right PCC/precuneus, right AG, and right MTG might be common and distinct NH patterns in MDD, and these brain regions might play important roles in the pathophysiology of MDD.

A number of previous studies have observed the associations between abnormal neural activity and clinical variables in MDD. So we hypothesized that there were some correlations between NH abnormalities and clinical variables in MDD. In the present study, we found that merely for melancholic patients, the NH value of the right MTG was negatively correlated with the TEPS total scores and TEPS contextual anticipatory scores. Anhedonia, as one of the typical symptoms of MDD, especially melancholic MDD, refers to decline in ability to experience pleasure [51]. Anticipatory anhedonia, associated with the future rewards of joyful feeling whereas consummatory anhedonia is associated with in-the-moment feelings of joy [52], can be captured by the Temporal Experience of Pleasure Scale (TEPS). In the Chinese version of TEPS, the anticipatory model was separated into two parts: contextual anticipatory and abstract anticipatory, and contextual anticipatory refers to more physical anticipating feelings of something more specific in nature [24]. So the result indicated that the abnormal NH of the right MTG in the melancholic MDD might be negatively associated with the severity of the anhedonia, especially the anticipatory anhedonia of concrete conditions.

A previous study has revealed that the ITG is involved in emotional processing and social cognition [46]. MDD exhibited lower NH [17] and higher ReHo [53] in the right ITG than healthy controls. In a previous study, melancholic MDD patients showed lower NH values in the right MTG as well as the temporal pole (MTG/TP) than healthy controls [11]. Although we could not directly compare these studies because of the illness durations, medication use, and different subtypes of MDD, these studies indicated the functional abnormalities of the temporal gyrus in MDD. Inconsistent with the above melancholic MDD study, we found lower NH values in the left ITG in the melancholic MDD patients compared with nonmelancholic MDD patients and healthy controls. In that study [11], researchers only compared the data between melancholic MDD patients and healthy controls. But in the present study, we also recruited nonmelancholic MDD patients. Thus, it might be more persuasive that the abnormal NH of the left ITG might be a distinct feature in the neurobiology of melancholic MDD.

The superior frontal gyrus, as an important part of the prefrontal gyrus, is involved in self-consciousness, emotion regulation, and cognitive processing [54, 55]. The medial prefrontal cortex (MPFC) engages in self-referential processing [56]. Lower coherence-based ReHo (Cohe-ReHo) in the bilateral frontal gyrus was observed in treatment-sensitive depression (TSD) [57]. Some previous studies have observed lower structural or functional changes in the prefrontal cortex in MDD [58–61]. Our present study has observed increased NH values in the bilateral SMFG in melancholic MDD patients compared with the other two groups, which is inconsistent with these previous studies. We could not compare them directly not only because of different methods and inclusive/exclusion criteria but also because our subjects were the melancholic MDD patients instead of all subtypes of MDD. Definitely, we highlighted the importance of bilateral SMFG in the melancholic MDD, and the abnormal NH of the bilateral SMFG might be a distinct feature in the neurobiology of melancholic MDD. To sum up, we could speculate that decreased NH in the left ITG and increased NH in the SMFG might be distinctive neurobiological features of melancholic MDD.

SVM has been widely applied in extensive biomedical applications for the diagnoses of MDD [61, 62], schizophrenia [63], and other psychiatric disorders [64]. The accuracy of using gray matter applying SVM to correctly distinguish between refractory depression (RDD) and nonrefractory depression (NDD) patients was 69.57% in a previous study [65]. The accuracy, sensitivity, and specificity of distinguishing pediatric patients with unipolar depression from healthy controls were 78.4%, 76.0%, and 80.8%, respectively [66]. SVM provided a specificity of 90.6% for distinguishing the MDD patients from healthy controls previously [61]. An eligible diagnostic indicator should have the sensitivity or specificity of no less than 60%, and greater than 70% is beneficial to establish diagnostic indicators [65, 67]. The present SVM results showed that the abnormal NH values in the combination region of the left SMFG and left ITG exhibited accuracy, sensitivity, and specificity of 79.66% 70.97%, and 89.29%, respectively, for distinguishing the patients with melancholic MDD from patients with nonmelancholic MDD. Hence, the NH values in the combination region of the left SMFG and left ITG could be employed as a potential imaging marker for distinguishing melancholic MDD patients from nonmelancholic MDD patients.

Our study has some limitations. First is the small sample size. Second, we recruited both melancholic and nonmelancholic MDD patients, but the further classification of nonmelancholic MDD is not clear due to the small sample size. Therefore, we were unable to further explore the neurobiological differences between different subtypes of nonmelancholic MDD.

## 5. Conclusion

Our present study is the first to compare the NH alterations in melancholic MDD patients and nonmelancholic MDD patients. Our findings showed the common and distinct network homogeneity patterns in MDD. Decreased NH in the left inferior temporal gyrus and increased NH in bilateral superior medial frontal gyrus may be distinctive neurobiological features of melancholic MDD. The NH values of the right middle temporal gyrus might be correlated with the clinical features in melancholic MDD. The NH values in the combination region of the left superior medial frontal gyrus and left inferior temporal gyrus may be a potential imaging marker to distinguish melancholic MDD and nonmelancholic MDD.

## Figures and Tables

**Table 1:** Demographic and clinical characteristics of the participants.

	Melancholic (*n* = 31)	Nonmelancholic (*n* = 28)	Healthy controls (*n* = 32)	*F*, *t*, or *χ*^2^ value	*p* value (two-tailed)
Age (years)	28.65 ± 5.30	32.04 ± 8.18	29.59 ± 5.00	2.291	0.107^a^
Gender(male/female)	10/21	10/18	15/17	1.55	0.461^b^
Handedness (right/left)	31/0	28/0	32/0		
Education(years)	15.16 ± 3.20	12.54 ± 3.00	14.59 ± 2.82	6.143	0.003^a^
Illness duration (months)	6.75 ± 4.26	5.96 ± 4.64		-0.68	0.500^c^
HRSD-17 scores	21.77 ± 3.79	21.00 ± 3.14	0.94 ± 0.95	527.891	<0.001^a^
BAI scores	44.00 ± 11.51	38.77 ± 9.84	22.63 ± 2.28	50.895	<0.001^a^
SHAPS-C scores	37.23 ± 6.04	31.89 ± 5.24	21.59 ± 5.36	64.191	<0.001^a^
TEPS total scores	58.30 ± 14.19	69.46 ± 11.16		-3.315	0.002^c^
TEPS abstract anticipatory	13.17 ± 4.79	17.04 ± 3.85		-3.373	0.001^c^
TEPS contextual anticipatory	13.13 ± 3.96	16.68 ± 3.64		-3.540	0.001^c^
TEPS abstract consummatory	20.20 ± 5.21	22.39 ± 5.28		-1.592	0.117^c^
TEPS contextual consummatory	11.80 ± 3.23	13.36 ± 3.27		-1.824	0.074^c^

HRSD-17: 17-item Hamilton Rating Scale for Depression; BAI: Beck anxiety inventory; SHAPS-C: the Chinese version of Snaith-Hamilton Pleasure Scale; TEPS: the Chinese version of the Temporal Experience of Pleasure Scale. ^a^The *p* value was obtained by analyses of variance. ^b^The *p* value was obtained by a chi-square test. ^c^The *p* value was obtained by two-sample *t*-tests.

**Table 2:** Significant DMN NH differences across groups.

Cluster location	Peak (MNI)			Number of voxels	*T* value
	*x*	*y*	*z*		
*Melancholic vs. healthy controls*					
Left inferior temporal gyrus	-48	-12	-27	32	-3.3543
Right middle temporal gyrus	45	3	-27	49	-3.9893
Left middle temporal gyrus	-63	-39	0	28	-2.9141
Left PCC/precuneus	-6	-48	15	30	-2.8359
Right superior medial frontal gyrus	15	60	3	22	2.6930
Left superior medial frontal gyrus	-9	57	9	23	3.1756
Right anterior cingulate cortex	12	42	12	22	2.4894
Right angular gyrus	60	-57	39	27	2.3859
Right PCC/precuneus	18	-51	33	48	3.6568
*Nonmelancholic vs. healthy controls*					
Right middle temporal gyrus	51	-18	-21	54	-3.1047
Left superior medial frontal gyrus	-6	60	33	36	-2.6479
Right superior medial frontal gyrus	6	57	36	28	-3.5704
Right PCC/precuneus	18	-54	33	41	3.7047
Right angular gyrus	54	-57	33	21	2.7685
*Melancholic vs. nonmelancholic*					
Left inferior temporal gyrus	-54	-15	-30	83	-3.6810
Left superior medial frontal gyrus	-9	57	6	38	3.1369
Right superior medial frontal gyrus	9	57	42	70	2.6833

DMN: default-mode network; NH: network homogeneity; MNI: Montreal Neurological Institute; PCC: posterior cingulate cortex.

## Data Availability

The data used in this study are available from Prof. Wenbin Guo upon request.
